# Major depressive disorder in Parkinson’s disease: a cross-sectional study from Sri Lanka

**DOI:** 10.1186/s12888-014-0278-8

**Published:** 2014-09-30

**Authors:** Tharini Ketharanathan, Raveen Hanwella, Rajiv Weerasundera, Varuni A de Silva

**Affiliations:** North Western Mental Health, Melbourne Health, Victoria, Australia; Department of Psychological Medicine, University of Colombo, Colombo, Sri Lanka; Department of Psychiatry, University of Sri Jayewardenepura, Nugegoda, Sri Lanka

**Keywords:** Parkinson’s disease, Depression, Prevalence, Sri Lanka

## Abstract

**Background:**

Depression is common in Parkinson’s disease (PD), and has a significant impact on the functional level of those affected. It is well studied in Western populations but data from Asia is limited. This study aims to estimate the prevalence of depression among PD patients attending a tertiary care outpatient clinic in Sri Lanka and identify potential risk factors.

**Methods:**

One hundred and four consecutive idiopathic PD patients as defined by the United Kingdom Parkinson’s Disease Society Brain Bank Diagnostic Criteria were recruited to the study. An interviewer administered questionnaire, the Hoehn-Yahr staging scale and the Schwab-England Activities of Daily Living Scale (SEADL) were used for assessment. Depression was diagnosed through a semi-structured clinical interview based on DSM-IV-TR criteria and all subjects were rated with the Montgomery-Asberg Depression Rating Scale (MADRS).

**Results:**

The prevalence of depression in the study population was 37.5%. Among the depressed 12 (30.8%) had mild depression, 21 (53.8%) moderate depression and 6 (15.4%) had severe depression. Depression was significantly associated with the stage of PD, functional impairment, civil status, educational level, caregiver dependence and concomitant diabetes mellitus.

**Conclusion:**

A significant proportion of PD patients suffers from depression. The prevalence rate of depression in the sample was similar to that reported in previous studies. Depression in PD is significantly associated with functional impairment.

## Background

Parkinson’s disease (PD) is the second commonest neurodegenerative disorder, its prevalence only being less than that of Alzheimer’s disease [1]. It affects approximately 1% of individuals older than 55 years [2]. Many patients with PD have clinically significant non-motor symptoms such as anxiety, depression, fatigue, sleep disturbance and sensory symptoms [3].

Up to 90% of patients with idiopathic PD experience psychiatric complications, including major depression [4]. Depression is the most common neuropsychiatric disturbance in PD [5] with its prevalence estimated to be around 35% [6]. It is also the most important predictor of poor quality of life in PD [1] and the most significant factor associated with experienced quality of life [7]. These findings emphasize the need to study the factors associated with depression in this population.

In comparison to the Western world there is a paucity of research on PD related psychiatric manifestations in Asia, especially in the South Asian region. Findings from Western populations cannot be directly extrapolated to the population of this region due to significant socio-cultural differences. This may also be supported by the observation that rates of depression have been shown to vary widely between different countries [1]. This underscores the importance of studying depression in PD in different countries and in a variety of socio-cultural contexts. To our knowledge, to date, only one study has been conducted in the South Asian region on this subject, which found a higher prevalence of depression, greater overall disability and inferior quality of life among PD patients compared to a control group of patients with other chronic medical illnesses [8]. The present study, the first of this nature in Sri Lanka, aims to estimate the prevalence of major depression in PD and identify potential risk factors in a local and regional context.

## Methods

The study was conducted in the outpatient Movement Disorder and Neurology clinics of the premier hospital in Sri Lanka, a tertiary care centre, over a period of 3 months. One hundred and four consecutive patients with idiopathic PD, satisfying the required number derived from sample size calculation and diagnosed according to United Kingdom Parkinson’s Disease Society Brain Bank Diagnostic Criteria (UK-PDS-BB) [9], were recruited to the study after excluding patients with severe speech difficulty. Clinical records of the selected patients were perused to establish the initial presentation and to understand the initial assessor’s view about the diagnosis.

Informed consent was obtained from the study participants. Ethical clearance was obtained from the Ethics Committee of National Hospital of Sri Lanka.

Data related to socio-demographics, PD and other health associated factors of the study subjects were recorded using an interviewer-administered questionnaire. The severity of PD was graded according to Hoehn-Yahr Staging system [10]. Functional impairment was estimated using the Schwab and England Activities of Daily Living scale (SEADL) [11]. The rating was done by the first author based on the information obtained from the patient and caregiver. A semi-structured clinical interview was conducted by the first author and major depression was diagnosed according to DSM-IV-TR criteria [12]. The DSM-IV-TR diagnosis of depressive disorder was considered the gold standard. Irrespective of the presence or absence of depression all participants were rated using the Montgomery-Asberg Depression Rating Scale (MADRS) [13]. The MADRS scores were utilized primarily to rate the severity of depression in those diagnosed with the disorder. The MADRS cut-off scores used to rate severity were ≤20: mild depression, 21–34: moderate depression and above 34: severe depression [14]. The MADRS was not translated into the local languages as it was scored by the investigators. While the interview was conducted in local languages, the assessments were made in English by the investigators. The investigators are bilingual and conduct clinical assessment interviews in the local languages and record data and management plans in English. The validation of the MADRS score using a structured clinical interview are reported in the results section.

### Statistical analysis of data

Statistical analysis included descriptive statistics, Pearson’s chi-square test, the Student’s independent *t*-test and univariate logistic and linear regression analyses. The Chi–square test examined the association between categorical variables. Logistic regression analysis calculated the odds ratio for each event in order to examine the possible association of demographic and clinical parameters with depression. Linear regression models were applied to assess the predictive power of variables such as age at assessment towards depression. The level of statistical significance was p < 0.05. All analyses were performed with the Statistical Package for Social Sciences (SPSS) version 16.0 for Windows.

## Results

### Description of socio-demographic variables

The socio-demographic data of the sample are reported in Table 1. The total number of study participants was 104. The mean age of the sample was 62.5 years (SD ± 9) (age range 44 to 83 years). The largest occupational group in the sample comprised of skilled workers, who numbered 35 (33.7% of the total sample). Occupational classes were assigned as defined by the Registrar-General Social Classes Classification [15].

**Table 1:** **Association between major depressive disorder and socio-demographic characteristics**

**Variables**	**Total no (%)**	**Depression**		**OR (95% CI)**	***p*** **value**
		**Yes (n,%)**	**No (n,%)**		
**Age category (years)**					
≤50	14(13.5%)	4(28.6%)	10(71.4%)	1	0.136
51-60	20(19.2%)	12(60.0%)	8(40.0%)	3.75(0.86-16.22)	
61-70	45(43.3%)	13(28.9%)	32(71.1%)	1.02(0.27-3.82)	
71-80	24(23.1%)	10(41.7%)	14(58.3%)	1.79(0.43-7.35)	
> 80	1(0.96%)	0(0.0%)	1(100.0%)	0	
**Gender**					
Male	64(61.5%)	23(35.9%)	41(64.1%)	1	0.677
Female	40(38.5%)	16(40.0%)	24(60.0%)	1.188(0.52-2.67)	
**Civil status**					
Single	10(9.6%)	2(20.0%)	8(80.0%)	1	0.039
Married	74(71.2%)	29(39.2%)	45(60.8%)	2.57(0.51-13.00)	
Separated/divorced	6(5.7%)	5(83.3%)	1(16.7%)	20.00(1.41-282.4)	
Widowed	14(13.5%)	3(21.4%)	11(78.6%)	1.09(0.14-8.12)	
**Level of education**					
Up to GCE O/L	81(77.9%)	35(42.7%)	47(57.3%)	3.35(1.04-10.78)	0.035
GCE A/L & higher	22(21.1%)	4(18.2%)	18(81.8%)	1	
**Currently employed**					
Yes	20(19.2%)	6(30.0%)	14(70.0%)	1	0.441
No	84(80.8%)	33(39.3%)	51(60.7%)	1.51(0.52-4.32)	
**Reason for unemployment**					
Never employed	1(1.2%)	0(0.0%)	1(100.0%)	0.00	0.433
Retired with age	25(29.7%)	7(28.0%)	18(72.0%)	0.45(0.11-1.83)	
Quit due to PD	45(53.6%)	20(44.4%)	25(55.6%)	0.93(0.27-3.22)	
Other reasons	13(15.5%)	6(46.2%)	7(53.8%)	1	
**Monthly income in LKR**					
≤10,000	33(31.7%)	16(48.5%)	17(51.5%)	2.04(0.78-5.36)	0.285
10,001-20,000	33(31.7%)	11(33.3%)	22(66.7%)	1.08(0.4-2.93)	
>20,000	38(36.5%)	12(31.6%)	26(68.4%)	1	

OR – Odds ratio; GCE O/L- General Certificate in Education Ordinary Level,

GCE A/L - General Certificate in Education Advanced Level; LKR -Sri Lankan Rupee,

### Prevalence of major depressive disorder

Thirty-nine subjects were diagnosed with a major depressive episode according to DSM-IV-TR criteria. Thus the prevalence of depression in this population was 37.5% (95% CI 28.04-46.96).

The MADRS scores ranged from 0 to 46. The mean MADRS score of the total sample was 13.14 (SD ± 11.81). In the depressed and non-depressed groups the mean MADRS scores were 26.08(SD ± 8.3) and 5.38(SD ± 4.5) respectively. Among the depressed, 12 (30.8%) had mild depression, 21 (53.8%) suffered moderate depression and 6 (15.4%) had severe depression, when categorised on the MADRS score.

### Major depressive disorder and socio-demographic characteristics

Unadjusted odds ratios obtained from logistic regression analysis are reported in Table 1. The mean age of the depressed, 61.87 years (SD ± 8.46), was not significantly different from that of the non-depressed individuals (62.92 years) (SD ± 9.4) (t = .571, p = .569). Age was not a significant predictor of depression in linear regression analysis.

### Description of clinical variables

The clinical data are shown in Table 2. Only two subjects reported a family history of mental illness. The majority of the 17 subjects who reported past psychiatric issues had substance related problems mainly in the form of dependence but were currently abstinent and 4 (3.8%) had a history of depressive episodes (data not shown in the table).

**Table 2:** **Association between major depressive disorder and Parkinson's disease and other clinical correlates**

**Variables**	**Total no (%)**	**Depression**		**OR (95% CI)**	***p*** **value**
		**Yes**	**No**		
**Age of PD onset (years)**					
<40	10(9.6%)	4(40.0%)	6(60.0%)	1	0.813
41-50	20(19.2%)	9(45.0%)	11(55.0%)	1.22(0.26-5.73)	
51-60	42(40.4%)	13(31.0%)	29(69.0%)	0.67(0.16-2.79)	
61-70	26(25.0%)	11(42.3%)	15(57.7%)	1.10(0.24-4.85)	
>70	6(5.8%)	2(33.3%)	4(66.7%)	0.75(0.09-6.23)	
**Duration of PD (years)**					
< 5	42(40.4%)	28(66.7%)	14(33.3%)	1	0.114
5 to 10	28(26.9%)	13(46.4%)	15(53.6%)	2.30(0.86-6.15)	
> 10	34(32.7%)	24(70.6%)	10(29.4%)	0.83(0.31-2.21)	
**Levodopa dose (mg/day)**					
< 250	15(14.4%)	4(26.7%)	11(73.3%)	1	0.8
250 - 500	41(39.4%)	16(39.0%)	25(61.0%)	1.76(0.47-6.49)	
500 - 750	22(21.2%)	10(45.5%)	12(54.5%)	2.29(0.55-9.47)	
750 - 1000	15(14.4%)	5(33.3%)	10(66.7%)	1.37(0.28-6.60)	
1000 - 1250	9(8.7%)	4(44.4%)	5(55.6%)	2.20(0.38-12.57)	
**Ropinirole treatment**					
Yes	21(20.2%)	11(52.4%)	10(47.6%)	10(47.6%)	0.115
No	83(79.8%)	28(33.7%)	55(66.3%)	55(66.3%)	
**Diabetes**					
Yes	12(11.5%)	9(75.0%)	3(25.0%)	6.2(1.56-24.5)	0.004
No	92(88.5%)	30(32.6%)	62(67.4%)	1	
**Significant chronic medical illness comorbid with PD**					
Yes	48(46.2%)	22(45.8%)	26(54.2%)	1.96	0.086
No	56(53.8%)	17(30.4%)	39(69.6%)	1	
**Hoehn-Yahr stage**					
I and II	24(23.1%)	2(8.3%)	22(91.7%)	1	0.003
III	55(52.9%)	27(49.1%)	28(50.9%)	10.61(2.27-49.53)	
IV and V	25(24.0%)	10(40.0%)	15(60.0%)	7.33(1.40-38.33)	
**Schwab-England score (%)**					
≥80	43(41.4%)	8(18.6%)	35(81.4%)	1	0.008
50-70	45(43.3%)	24(53.3%)	21(46.7%)	5.00(1.90-13.13)	
30-40	12(11.5%)	5(41.7%)	7(58.3%)	3.12(0.78-12.43)	
≤20	4(3.8%)	2(50.0%)	2(50.0%)	4.37(0.53-35.90)	
**Caregiver**					
Independent	25(24.0%)	5(20.0%)	20(80.0%)	1	0.038
Dependent	79(76.0%)	34(43.0%)	45(57.0%)	3.02(1.03-8.86)	

OR – Odds ratio.

The longest duration of PD encountered was 26 years. In addition to the commonly used anti-Parkinsonian medications such as levodopa (n = 102, 98.1%), benzhexol (n = 96, 92.3%), amantadine (n = 3, 2.9%) and ropinirole (n = 21, 20.2%), patients were also receiving medications such as bromocriptine, madopar, clonazepam and propronolol. Two subjects (1.9%) were treated only with benzhexol. Sixty-seven (64.4%) were on 2 anti-Parkinsonian medications, with over 90% of this group receiving the benzhexol and levodopa combination, while 23.1% were on a combination of 3 medications. Thirty-nine patients (37.5%) were on cardiotropics, antihypertensives or oral hypoglycaemics.

Diabetes mellitus (n = 12, 11.5%), hypertension (n = 29, 27.9%), heart disease (n = 8, 7.7%), epilepsy (n = 2, 1.9%) and stroke (n = 1, 0.96%) were significant chronic medical illnesses concomitant with PD in the sample.

### Major depressive disorder and Parkinson’s disease correlates

Table 2 shows the association between major depressive disorder and PD and other clinical correlates. Dependence on a caregiver was determined by accounts from patients and accompanying relatives or friends with specific questions focusing on activities of daily living. Among the 79 caregiver dependent patients, 69 (87.3%) were cared by the immediate family, 6 (7.6%) by the extended family and 3 (3.8%) by non-relatives.

Only eight of the 39 depressed subjects were receiving anti-depressants at the time of assessment. The most frequently prescribed anti-depressant was amitriptyline (average dose ≤25 mg/day).

### Major depressive disorder and level of functioning

Using the SEADL scale, the patients’ level of functioning was rated by the investigator. About 41% of patients had retained 80% function, which corresponds to almost independent functioning despite slowness resulting in doubling of time for completing activities. The mean SEADL score among the depressed (60.51%) was significantly lower than that of the non-depressed (69.69%) (t = 2.289, p = 0.024).

### Sensitivity and specificity of MADRS

As the MADRS scores were available for all patients, *post hoc* we decided to determine the sensitivity and specificity of the MADRS as a diagnostic tool for DSM-IV-TR diagnosis of major depressive disorder in PD. In a previous study, a MADRS score ≥18 had been identified as a good diagnostic cut-off in PD populations [16]. At this cut-off MADRS showed a sensitivity of 89.7% and a specificity of 100% when applied to our data (Table 3).

**Table 3:** **Sensitivity and specificity of MADRSi**

		**DSM-IV-TR**		
		**Depressed**	**Not depressed**	**Total**
MADRS (≥18)	Depressed	35	0	35
	Not depressed	4	65	69
	Total	39	65	104

MADRS - Montgomery-Asberg Depression Rating Scale.

DSM-IV-TR - Diagnostic and Statistical Manual of Mental Disorders, 4th Edition, Text Revision.

## Discussion

This study attempted to determine the prevalence of depression and identify potential risk factors in the PD population in Sri Lanka. The prevalence of major depressive disorder in the study population was 37.5%, similar to the rates reported in most Western populations [3,5,6]. The following factors had significantly increased the odds of developing depression: Hoehn-Yahr stage, functional impairment, civil status, level of education, caregiver dependence and concomitant diabetes mellitus. More than half of the depressed subjects were moderately depressed. Evidence suggests depression is usually milder in this population [17,18].

In comparison to the majority of studies done in Asia which utilized assessment scales for the diagnosis of depression, the present study used a semi-structured DSM-IV-TR based clinical interview for this purpose. A study from Taiwan [19], which also used an interview based assessment, reported a frequency of major depressive disorder of 16.5%, and major and other depressive disorders, taken together, of 42.2%.

Studies using rating scales to diagnose depression report a higher prevalence than those which use a structured clinical interview and diagnostic criteria [1,20]. A study from India using MADRS and the Beck Depression Inventory (BDI) found the rates as 54% and 49% respectively [8]. Other studies have reported rates of depression of 46% from the Philippines using the MADRS [21], 56% from Japan using the BDI-II [22] and BDI [23] and 65% using the Zung Self-Rating Depression Scale [24] and 21% in China using the Hamilton Rating Scale for Depression [25]. Interestingly in another study from Japan among the 38% identified as depressed by BDI-II, nearly 75% did not show depressed mood [26].

We could not find an association between major depression in PD and age, gender, employment status, occupational class, reasons for unemployment, income, past psychiatric history, age of PD onset, duration of PD, levodopa dose, ropinirole treatment or the presence of concomitant other major medical illnesses. Among the anti-Parkinsonian agents ropinirole was given special consideration as it is commonly used to treat disabling dopa induced dyskinesia [27,28], but also appears to ameliorate depressive symptoms in PD [27].

The prevalence of depression was higher in Hoehn-Yahr stages III, IV and above. Similar findings have been reported previously [2,17]. It was highest in stage III possibly because this is the stage where the debility becomes marked. Though the rise in the rate of depression would be expected to be linear as the disability increases this was not observed. This is possibly due to the inability of the severely disabled to physically attend the clinic resulting in a lesser number of patients in severe stages participating in the study which may have influenced the results. Existing views about the association between the severity of PD and depression are contradictory: some studies reported it as positive [2,17,19], while others showed no association [29].

The putative association between functional impairment and depression has been a source of debate for long. Depression has been commonly found to be correlated with increased impairment in activities of daily living [2,17,19,20] and self-rated disability has been identified as the greatest predictor of depressive symptoms [29]. The relationship between depression and functional impairment in PD may be reciprocal.

Our results indicate that depression in PD is significantly associated with a state of dependence in the affected population. There could be two explanations for this: while dependence due to physical debility can give rise to depression, depression with low motivation and reduced activity could also make people dependent on others. Among the large number of patients who were caregiver dependent all but one had informal caregivers, which highlights the customs of Sri Lankan society where most of its elderly and feeble members are cared for within a close family network, without being institutionalized.

The association between diabetes mellitus and depression in PD is an important finding from this study, which has not been widely reported. Depression and diabetes commonly co-occur in the general population with rates of depression twice as high in people with diabetes compared to those without [30]. The rate of depression among PD patients with diabetes in our study is 75%. Despite the small number of patients in this group this very high rate merits further exploration regarding the possible mechanisms underlying this comorbidity.

About 30% of our study population were categorised under early-onset PD (onset ≤ age 50) and about 10% developed the illness before 40 years. Most studies reported rates between 3%-5% for an onset ≤ 40 years, while in Japan higher rates, up to 10%, were reported [31]. It would appear that the rates observed in our study are elevated in comparison to Western studies. We speculate that the complexity of early onset PD leads to increased referrals to tertiary care centres, like our study centre; hence the over representation. However, a distinct type of PD prevalent among Asian populations is also a possibility, given the high rates noted in the Japanese study.

Only 20% of depressed subjects were on antidepressants at the time of assessment which draws attention to how depression is poorly identified and treated in neurology settings. The actual number treated might have been lower as it is possible that low dose antidepressants were prescribed for chronic pain instead of depression in some patients. Depression in PD is commonly found to be under-recognized and under-treated [3,5].

Overall our findings are in keeping with what is reported in Western populations, in relation to prevalence and associated factors of depression in PD. Our study population showed some unique or less commonly reported characteristics such as a higher proportion of early onset PD and association of depression with diabetes. These characteristics merit further inquiry to ascertain whether they are replicated regionally and globally. Further, the differences in depression between cultures may be in the expression, and studying the phenomenology of depression in PD across cultures maybe useful.

### Strengths and limitations

A major strength of this study is using a diagnostic criteria based clinical interview as the gold standard for diagnosing depression which was not commonly employed in previous studies. The limitations were the sample being out-patient and largely urban and therefore possibly not being representative of the general population, the modest sample size which could have led to the wide confidence intervals in some results, the potential confounding effect of subtle cognitive impairment on the presentation and assessment of depression and hospital based sampling likely to be yielding a higher prevalence of depression, as community based studies tend to find lower rates [1,20,32].

The implications for practice from the study are the need for greater awareness among clinicians regarding depression in PD, its early detection and the need for intervention. Larger, better designed studies are warranted to conclusively study and identify varying psychiatric manifestations associated with PD.

## Conclusion

In conclusion this first study in Sri Lanka on PD population found that a significant proportion of these patients suffer from major depression. Depression was significantly associated with a number of socio-demographic and clinical factors including PD stage and functional impairment; and was predominantly found in mild to moderate severity. In addition, the study population displayed some unique features such as depressive vulnerability in the presence of diabetes and a higher proportion of early onset PD. Whether there is a regional effect in these findings and possible underlying mechanisms for them need further exploration.

## Abbreviations

BDI: Beck depression inventory
DSM-IV-TR: Diagnostic and statistical manual of mental disorders, 4th edition, text revision
GCE A/L: General certificate in education advanced level
GCE O/L: General certificate in education ordinary level
MADRS: Montgomery-asberg depression rating scale
PD: Parkinson’s disease
SEADL: Schwab and England activities of daily living scale
